# Identification of a Hypoxia-Associated Signature for Lung Adenocarcinoma

**DOI:** 10.3389/fgene.2020.00647

**Published:** 2020-06-23

**Authors:** Zhuomao Mo, Ling Yu, Zhirui Cao, Hao Hu, Shaoju Luo, Shijun Zhang

**Affiliations:** Department of Traditional Chinese Medicine, The First Affiliated Hospital, Sun Yat-sen University, Guangzhou, China

**Keywords:** lung adenocarcinoma, hypoxia, prognostic factor, signature, TCGA

## Abstract

**Background:**

A hypoxia microenvironment plays a role in the initiation and progression of many cancer types, but its involvement in lung adenocarcinoma is still unclear. This study aimed to explore the potential correlation between hypoxia and lung adenocarcinoma and establish the hypoxia-associated gene signature in lung adenocarcinoma.

**Methods:**

Lung adenocarcinoma cases were retrieved from The Cancer Genome Atlas (TCGA) and the Gene Expression Omnibus (GEO) databases. The genes to be included in the hypoxia-associated signature were selected by performing univariate Cox regression analysis and lasso regression analysis. Then, the gene signature was verified by performing a survival analysis and constructing the multiple receiver operating characteristic (ROC) curve. The CIBERSORT tool was then used to investigate the potential correlation between the gene signature and immune cells. Moreover, a nomogram was constructed and evaluated by calculating the C-index.

**Results:**

Four genes (*XPNPEP1*, *ANGPTL4*, *SLC2A1*, and *PFKP*) were included in the final signature. The results showed that patients in the high-risk group showed worse survival than those in the low-risk group. Moreover, we found two types of immune cells (memory activated CD4^+^ T cell and M0 macrophages) which showed a significant infiltration in the tissues of the high-risk group patients.

**Conclusion:**

The hypoxia-associated gene signature established and validated in this study could be used as a potential prognostic factor in lung adenocarcinoma and may guide the immunotherapy choice.

## Introduction

Lung cancer is estimated to be the most commonly diagnosed cancer in the world and the leading cause of cancer death (Bray et al., 2018). Lung adenocarcinoma is the most prevalent non-small cell lung cancer subtype (Coudray et al., 2018), comprising 50% of all lung cancer cases (Denisenko et al., 2018). About 70% of the patients present local progression or the presence of metastasis at the time of diagnosis (Molina et al., 2008). Moreover, lung adenocarcinoma is associated with a poor average 5-year survival rate of 18% (Imielinski et al., 2012). Therefore, there is an urgent need to develop an effective prognostic biomarker to predict this cancer type.

Hypoxia is a typical characteristic observed in the tumor microenvironment and drives the aggressiveness of many tumors, such as hepatocellular carcinoma (Kung-Chun Chiu et al., 2019), colorectal cancer (Qureshi-Baig et al., 2019), and esophageal squamous cell carcinoma (Zhang et al., 2019). Under hypoxic conditions, many transcription factors are activated in tumor cells, inducing various downstream signals regulating cell proliferation, motility, and apoptosis (Semenza, 2012). Genes correlated with the hypoxic status have been reported to have a prognostic prediction value. For example, overexpression of LBH under hypoxic conditions in gliomas was associated with poor prognosis (Jiang et al., 2019). In lung adenocarcinoma, *NLUCAT1*, a transcript that is strongly upregulated upon hypoxia, was proved to have significant prognostic value (Moreno Leon et al., 2019). Furthermore, *TRB3*, which is significantly upregulated by hypoxia, was correlated with a worse prognosis in lung adenocarcinoma patients (Cao et al., 2020).

Different models for predicting the prognosis of lung adenocarcinoma have been developed, based on the tumor microenvironment (Yue et al., 2019), *TP53* mutation (Long et al., 2019), and tumor immunology (Luo et al., 2019). However, to our knowledge, in lung adenocarcinoma, no signature based on hypoxia-associated genes has been constructed yet. Therefore, in this study, we constructed a signature and a nomogram based on hypoxia-associated genes, to explore the potential prognostic value of hypoxia-associated genes in lung adenocarcinoma patients.

## Materials and Methods

### Data Collection

The gene expression data of level 3 RNA-seq FPKM dataset and GSE68465 dataset were retrieved from The Cancer Genome Atlas-Lung adenocarcinoma (TCGA-LUAD), and the Gene Expression Omnibus (GEO) database, respectively. Furthermore, the related clinical data of the two datasets were retrieved. The list of hypoxia-associated genes was obtained from the hallmark gene sets of the Molecular Signatures Database^1^. A total of 200 genes were included in the analysis, all responding to low oxygen levels (Supplementary Appendix S1). The GSE72094 dataset obtained from the GEO database was used as a reference to validate the established signature.

### Identification of Differentially Expressed Genes in Two Datasets

The differential expression of the 200 hypoxic genes in the two datasets was analyzed with the R studio software, using the “limma” package. In all the analyses, a *P*-value < 0.05 was considered statistically significant. Genes were selected for further prognostic analysis only if they were differentially expressed in both datasets.

### Construction and Validation of the Hypoxic Gene Signature

After the selection of the differentially expressed genes, we identified the prognosis-related hypoxic genes by performing univariate Cox regression analysis and lasso regression analysis. A *P*-value < 0.05 in univariate Cox regression analysis was considered statistically significant, while the genes were considered eligible for lasso regression analysis only if they showed significance in the Cox regression analysis of both datasets. Then, we performed a lasso regression analysis for selecting reliable predictors. In this analysis, a lasso penalty was applied, to simultaneously accounting for shrinkage and variable selection. The optimal value of the lambda penalty parameter was defined by performing 10 cross-validations. After that, we selected the composition of the final gene signature and used it to generate the risk score according to the following formula:

Risk⁢score=(coefficient⁢mRNA1×expression⁢of⁢mRNA1)

     +(coefficient⁢mRNA2×expression⁢of⁢mRNA2)

     +…+

     (coefficient⁢mRNAn×expression⁢mRNAn)

The cases were divided into two groups (high risk or low risk), according to the risk score median and then the relationship between risk score and clinical data was investigated.

### Gene Signature Prognostic Independence Analysis

Due to missing entries in the clinical data available in the two datasets, we only kept the patient that had the following complete data: for the TCGA dataset, overall survival (OS), age, gender, T (the size of tumor), M (regional lymph node metastasis), N (distant metastasis), and stage; for the GEO dataset, OS, age, gender, and T. The survival analysis, exploring the time-dependent prognostic value of our gene signature, was performed using the “survival” package in the R Studio software. A *P*-value < 0.05 was considered statistically significant. The relationship between signature and other clinical data was also evaluated and visualized with a heatmap. Moreover, a multiple receiver operating characteristic (ROC) curve was performed to verify the prognostic accuracy. Besides, the lasso regression analysis, survival analysis, and ROC curve were performed again using a test dataset for validation.

### Relationship Between Signature and Immune Cell

To explore the potential relationship between the gene signature and immune cells in the context of lung adenocarcinoma, the mRNA expression matrix was normalized and the CIBERSORT tool was used to estimate the content of 22 human immune cells. Then, we divided the cases into two groups according to the median risk score and used the “vioplot” package in R Studio to visualize the data. A *P*-value < 0.01 was considered statistically significant.

### Predictive Nomogram Design

A predictive nomogram based on age, sex, stage, and risk score was built using the “rms” package and Cox regression model. Then, we used the Harrell’s concordance index (C-index) to evaluate the discrimination power of the nomogram, based on a bootstrap method using 1000 replicates. Furthermore, univariate and multivariate Cox regression analysis were performed to verify whether the signature was an independent prognostic factor.

## Results

### Selection of Hypoxic Genes and Construction of the Signature

**FIGURE 1:**
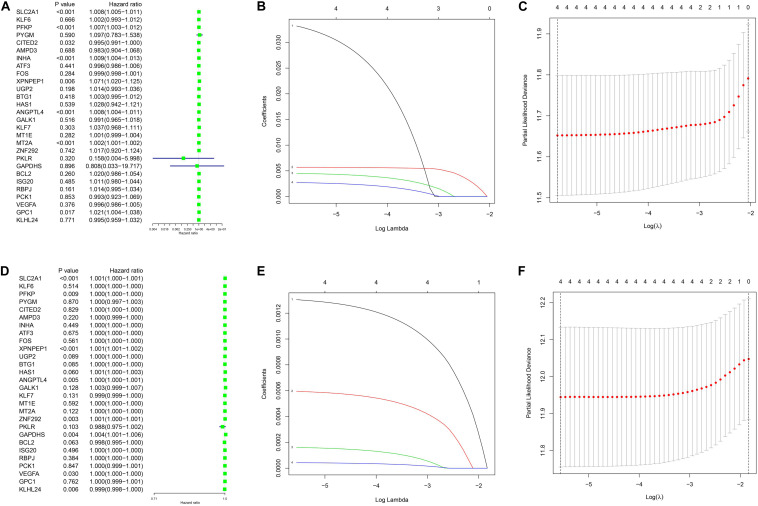
Univariance Cox regression and lasso regression results. **(A–C)** The TCGA-LUAD cohort results. **(D–F)** The GSE68465 cohort results. **(A,D)** show the univariance Cox regression analysis. **(B,E)** show the lasso regression analysis. **(C,F)** show the partial likelihood deviance for the lasso regression.

The clinical data details of two train datasets used in this study are shown in Table 1. Differential expression analysis was performed on 200 hypoxic genes, and the results showed that 155 genes and 34 genes were significantly different in the TCGA cohort and the GEO cohort, respectively. Heatmaps show the levels of the differentially expressed genes in detail (Supplementary Appendices S2, S3). A total of 28 hypoxic genes were selected for univariate Cox regression analysis because they were significantly differentially expressed in both cohorts. As illustrated in Figures 1A,D, eight genes (*SLC2A1*, *MT2A*, *ANGPTL4*, *INHA*, *PFKP*, *XPNPEP1*, *GPC1*, and *CITED2*) in the TCGA cohort and eight genes (*XPNPEP1*, *SLC2A1*, *ZNF292*, *GAPDHS*, *ANGPTL4*, *KLHL24*, *PFKP*, and *VEGFA*) in the GEO cohort were significantly different (*P* < 0.05). We then chose the four genes (*XPNPEP1*, *ANGPTL4*, *SLC2A1*, and *PFKP*) who were significantly different in both cohorts and performed a lasso regression analysis. The results of the lasso regression analysis (Figures 1B,C,E,F) further confirmed the signature composed of four genes: *XPNPEP1*, *ANGPTL4*, *SLC2A1*, and *PFKP*. The coefficients of the four chosen genes calculated by lasso regression analysis are shown in Table 2.

**TABLE 1:** Baseline patient characteristic.

**Variable**	**Number (*N*)**	
	**TCGA-LUAD cohort**	**GSE68465 cohort**
Age	19 patients missing	0 patients missing
<60	136	128
≥60	331	315
Gender	0 patients missing	0 patients missing
Male	222	223
Female	264	220
Stage	Eight patients missing	NA
I	262	–
II	112	–
III	79	–
IV	25	–
T	Three patients missing	Two patients missing
T1	163	150
T2	260	251
T3	41	28
T4	19	12
M	Four patients missing	NA
M0/MX	458	–
M1	24	–
N	One patient missing	NA
N0/NX	323	–
N1	90	–
N2	70	–
N3	2	–

*NA = not applicable.*

**TABLE 2:** Coefficients of 4 selected hypoxic genes.

**Gene**	**Coefficient (TCGA-LUAD)**	**Coefficient (GSE68465)**
XPNPEP1	0.033204	0.001304
SLC2A1	0.005672	0.000595
ANGPTL4	0.004505	0.000160
PFKP	0.002714	0.000042

### Relationship Between the Gene Signature and Clinical Parameters

**FIGURE 2:**
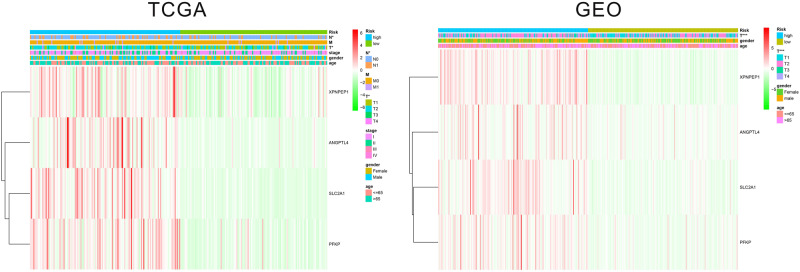
Heatmap of the hypoxic signature with other clinical parameters.

**FIGURE 3:**
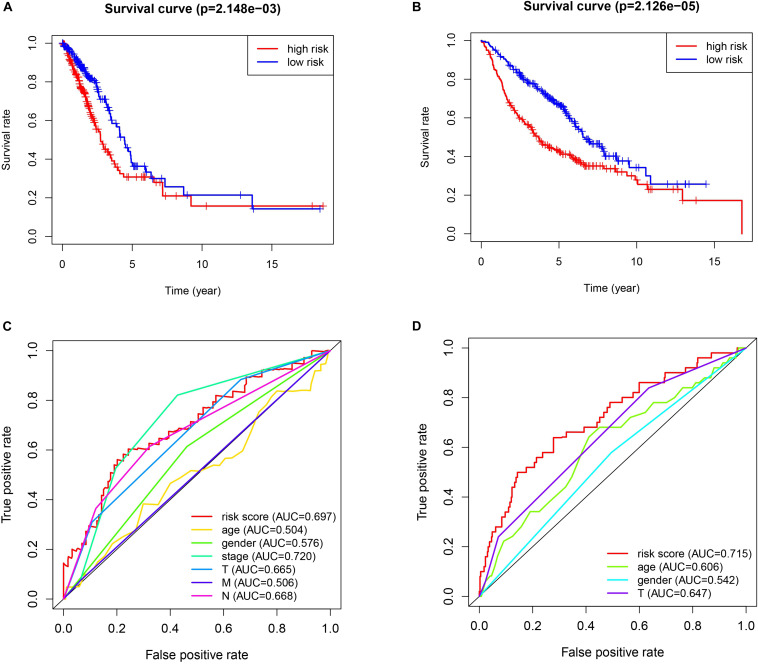
Survival analysis and multiple ROC curve results. **(A,C)** The TCGA-LUAD cohort results. **(B,D)** The GEO cohort results. **(A,B)** show the survival analysis. **(C,D)** show the multiple ROC curve.

The patients in the two datasets used to build the gene signature were divided into two risk groups, based on the median of risk score. All the genes included in the signature were highly expressed in the high-risk group and lowly expressed in the low-risk group, in both cohorts. Significant differences were found for tumor size and the presence of distant metastasis between the high-risk and low-risk groups in the TCGA cohort, and only for tumor size in the GEO cohort (Figure 2). The survival analysis found a significant difference between the two groups in both cohorts (Figures 3A,B). Furthermore, the multiple ROC curve plot (Figures 3C,D) demonstrated that our established signature has a certain predictive prognostic value (0.697 and 0.715 in the TCGA cohort and the GEO cohort, respectively).

### Validation in the Test Dataset

**FIGURE 4:**
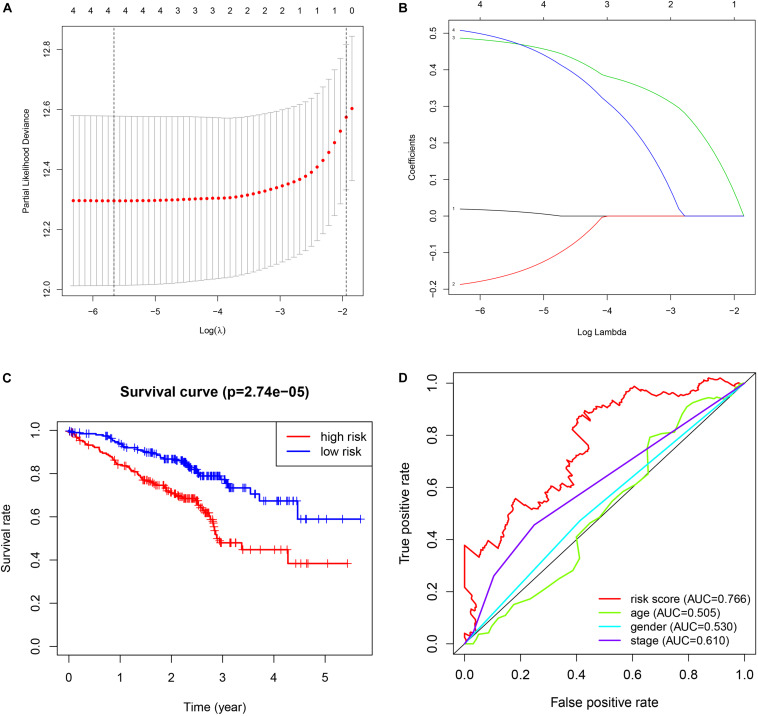
Validation of GSE72094 cohort. **(A)** The lasso regression analysis. **(B)** The partial likelihood deviance for the lasso regression. **(C)** The survival analysis. **(D)** The multiple ROC curve.

The validation results are presented in Figure 4. The procedures were the same as the train dataset. The signature was constructed using lasso regression analysis and the relevant results were shown in Figures 4A,B. The results of survival analysis (Figure 4C) indicated that significant difference was found between two groups. Furthermore, the ROC curve plot (Figure 4D) demonstrated that the established signature has the highest predictive prognostic value among the clinical parameters (AUC = 0.766).

### Relationship Between the Gene Signature and Immune Cell

**FIGURE 5:**
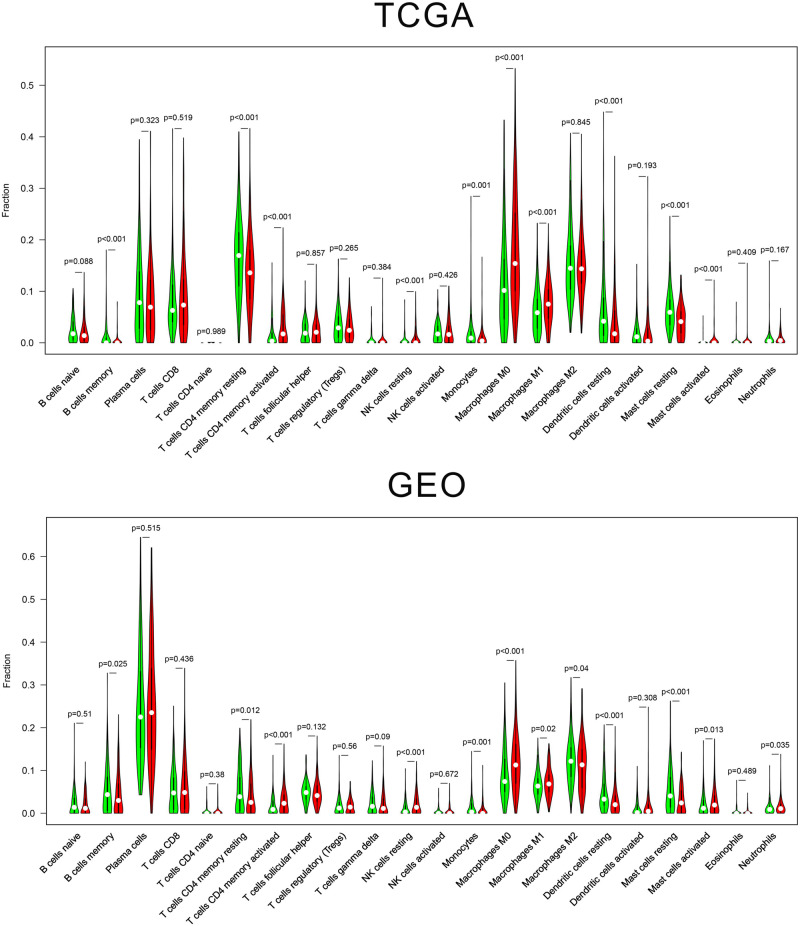
Vioplot of 22 immune cells content in the high-risk and low-risk group.

We estimated the presence of 22 immune cell types in our cohorts (Figure 5). In the TCGA cohort, 204 cases in the low-risk group and 209 cases in the high-risk group showed a significant difference for the presence of 10 immune cells types (memory B cells, memory-resting CD4^+^ T cells, memory-activated CD4^+^ T cells, resting NK, monocytes, M0 macrophages, M1 macrophages, resting dendritic cells, resting mast cells, and activated mast cells). In the GEO cohort, 221 cases in the low-risk group and 221 cases in the high-risk group showed a significant difference for the presence of six immune cells types (T cells CD4 memory activated, NK cells resting, monocytes, macrophages M0, dendritic cells resting, and mast cells resting).

### Predictive Nomogram Construction

**FIGURE 6:**
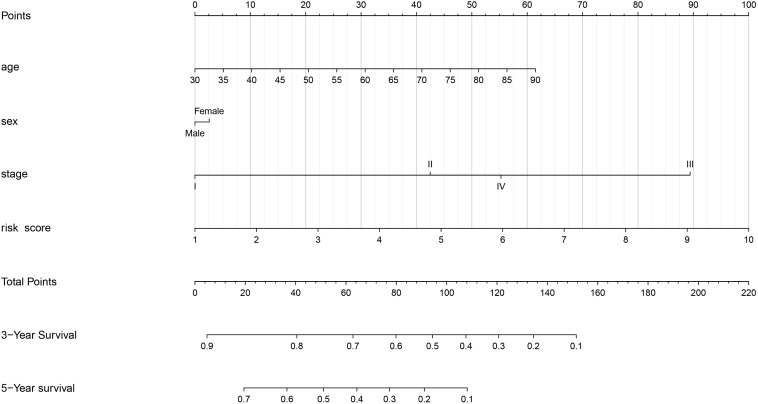
Nomogram of the gene signature.

**FIGURE 7:**
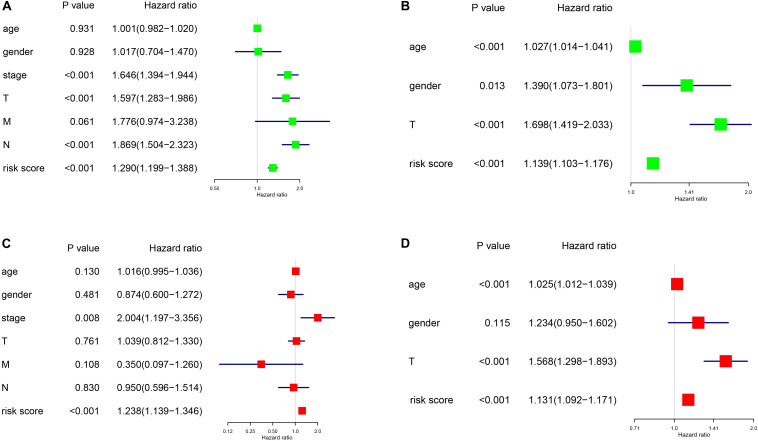
Univariance and multivariance Cox regression analysis of the gene signature. **(A,C)** The TCGA-LUAD cohort results. **(B,D)** The GSE68465 cohort results. **(A,B)** were univariance Cox regression analysis. **(C,D)** show the multivariance Cox regression analysis.

As shown in Figure 6, we constructed a nomogram to predict the 3-year and 5-year OS for patients with lung adenocarcinoma. The C-index of the nomogram was 0.726 (se = 0.034), supporting the nomogram suitability to predict the survival rate for lung adenocarcinoma. In addition, we performed univariate and multivariate Cox regression analysis, and the results revealed that the risk score calculated in our study can be used as an independent prognostic factor in lung adenocarcinoma in both cohorts (*P* < 0.01) (Figure 7).

## Discussion

Many hypoxia-associated genes have been reported to be correlated to lung adenocarcinoma (Moreno Leon et al., 2019; Cao et al., 2020). Moreover, the use of mRNA gene signatures based on certain tumor microenvironment characteristics showed good prediction potential in lung adenocarcinoma patients. In this study, we built a novel hypoxia-associated signature composed of four genes, using the expression data of two TCGA and GEO datasets. We used two train datasets, which contained more than 900 cases of lung adenocarcinoma, to obtain more reliable results than those obtained by using a single dataset. The genes included in the signature were chosen by the intersection of the differential expression analysis, univariate Cox regression analysis, and lasso regression analysis of the two datasets, demonstrating that eligible genes have a certain prognostic value in lung adenocarcinoma. Using the signature to divide the cohort into two risk groups, we found significant differences in OS and tumor size. The association between tumor size and hypoxia is worthy of notice: previous studies also reported a relationship between tumor size and hypoxia in cancer (Yang et al., 2019; Yu et al., 2019). Yang et al. found that, in hepatocellular carcinoma, hypoxia-induced EIF3J-AS1 expression, and the expression of EIF3J-AS1 was correlated with tumor size. Yu et al. found that, in prostate cancer, overexpression of HIF1α by HIF1α-M could increase tumor size. Our research further supports a correlation between tumor size and hypoxia in lung adenocarcinoma, but further studies are needed to investigate the potential mechanism involved. Moreover, the univariate and multivariate Cox regression analysis performed on both datasets showed the prognostic independence of our novel signature, further supporting the idea that hypoxia-associated genes affect the prognosis of lung adenocarcinoma, even though the mechanism underlying this relation is still unclear. Based on this signature, we constructed a predictive prognostic nomogram model, which could help in drafting a short-term therapeutic strategy for patients.

Previous studies investigated the relationship between hypoxia and immune cells in lung adenocarcinoma. Li et al. (2019) showed that a GBE1-mediated blockade recruits CD8^+^ T lymphocytes to the tumor microenvironment via the IFN-I/STING signaling pathway, with concurrent upregulation of PD-L1 in LUAD cells. Another study demonstrated that EML4-ALK enhances PD-L1 expression in lung adenocarcinoma via the HIF-1α and STAT3 pathways (Koh et al., 2016). To further explore the potential relationship between hypoxia and immune cells, we analyzed the differences in immune cells content between high-risk and low-risk groups in the two cohorts. Interestingly, we found that two immune cell types (memory-activated CD4^+^ T cells and M0 macrophages) significantly infiltrated in the high-risk group patients. At the moment, only macrophage cells (Zhang et al., 2014), CD8^+^ T cells (Li et al., 2019), and NK cells (Terry et al., 2017) have been correlated with hypoxia in lung adenocarcinoma. Tissue hypoxia has been considered as a key factor for tumor aggressiveness and metastasis. When hypoxic stress is maintained for a prolonged period, the observed phenotypic changes are especially exacerbated. It has been demonstrated that hypoxia stress-induced phenotypic diversity along the epithelial–mesenchymal transition (EMT) spectrum (Terry et al., 2017). Recent studies employing patients cohorts have indicated that human tumors with high EMT present higher immune activation marks and increased immune cell infiltration (Lou et al., 2016; Mak et al., 2016). Another underlying mechanim linking hypoxia, immune response, and lung adenocarcinoma is the GBE1 protein, which is downstream of the HIF1 pathway in hypoxia-conditioned lung cancer cells (Li et al., 2017). GBE1 can upregulate CCL5 and CXCL10 expression through STING signaling to activate the IFN-I pathway and potentiate T cell infiltration in the tumor (Li et al., 2019). Our research revealed a novel potential target for immunotherapy in lung adenocarcinoma (memory-activated CD4^+^ T cells) but more studies are needed to validate our discovery.

To our knowledge, this is the first study that constructed a hypoxia-associated signature and nomogram in lung adenocarcinoma. Differing from previous studies, our study focused on hypoxia-responding genes and investigated the potential relationship between hypoxia and clinical data, and hypoxia and immune cells. Moreover, the use of the intersection results on two different datasets and the validation of the third independent dataset enabled a rigorous screening process and the identification of a reliable signature. However, our methodology has some drawbacks. First, using a single characteristic (response to hypoxia) to establish the predictive model is an intrinsic weakness. Indeed, many other mechanisms influence the development and progression of lung adenocarcinoma. Second, whether hypoxic genes were particularly correlated to lung hypoxia was still unclear. Further studies are necessary to further confirm the role of hypoxic genes from different tissues. Third, only two of the genes included in the signature, *SLC2A1* (Martínezterroba et al., 2018) and *PFKP* (Lee et al., 2016) have previously been evaluated as prognostic markers for lung adenocarcinoma. Thus, more studies are needed, using independent cohorts and functional experiments to further validate the prognostic value of our signature and to shed light on the mechanism linking hypoxia and lung adenocarcinoma progression.

## Conclusion

In this study, we established for the first time a hypoxia-associated gene signature that could be used as a potential prognostic factor in lung adenocarcinoma and may guide the adoption of immunotherapy for suitable patients.

## Data Availability Statement

The datasets generated during the current study are available in the TCGA database (https://portal.gdc.cancer.gov/) and GEO database (https://www.ncbi.nlm.nih.gov/geo/).

## Author Contributions

ZM and SZ designed the manuscript. ZM and LY wrote and completed the manuscript. HH, SL, and ZC completed the data download and analysis. All the authors approved the final manuscript.

## Conflict of Interest

The authors declare that the research was conducted in the absence of any commercial or financial relationships that could be construed as a potential conflict of interest.

## Footnotes

1 http://software.broadinstitute.org/gsea/msigdb/index.jsp
