# Supplementary figures and images for: Identification of a Hypoxia-Associated Signature for Lung Adenocarcinoma
Source: Front Genet. 2020 Jun 23;11:647. doi: 10.3389/fgene.2020.00647 (PMC7324800; doi:10.3389/fgene.2020.00647)

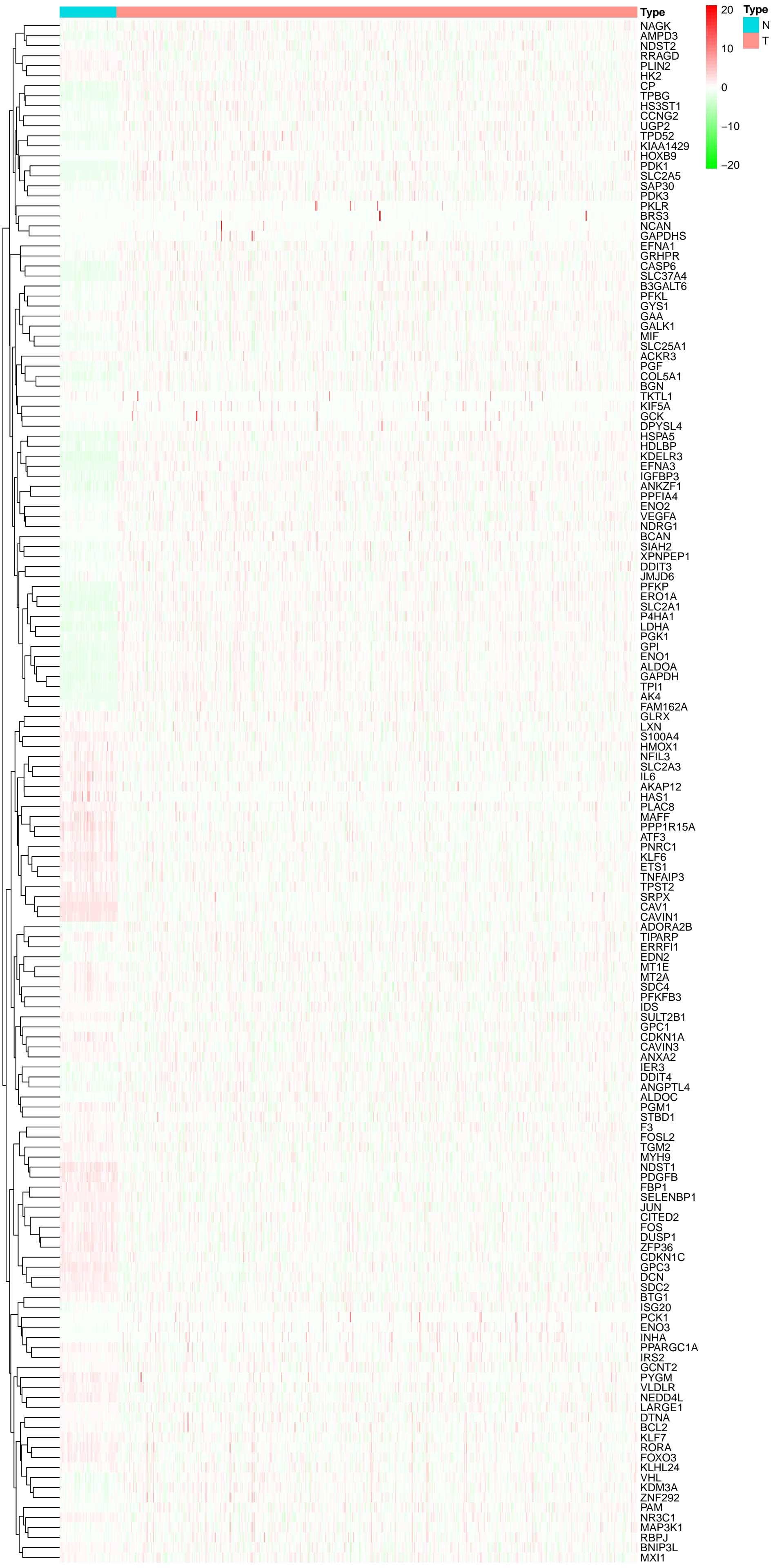

Supplement: Supplementary file 1 [file Data_Sheet_1.PDF]

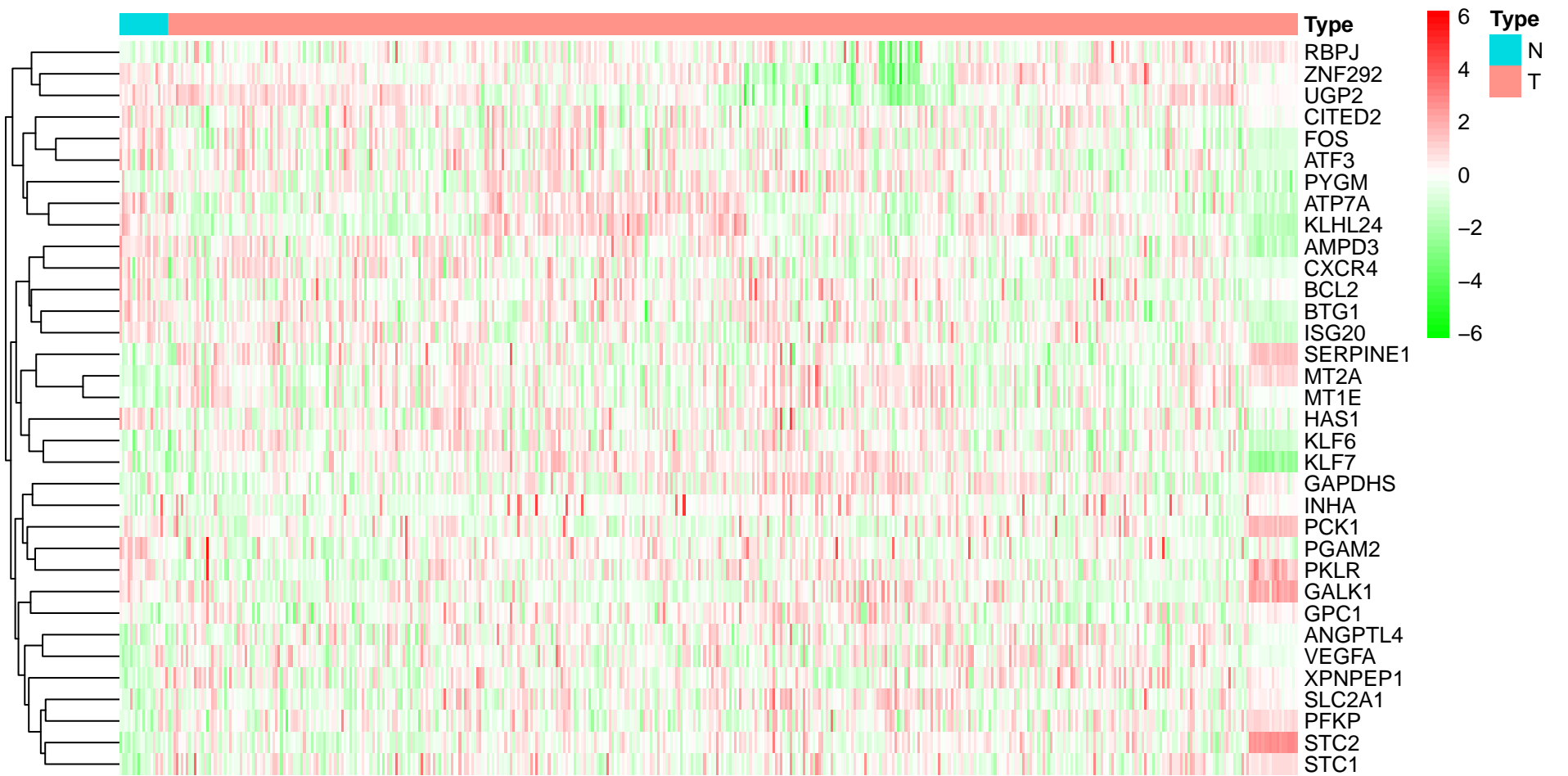

Supplement: Supplementary file 2 [file Data_Sheet_2.PDF]
